# A Continuing Educational Program Supporting Health Professionals to Manage Grief and Loss

**DOI:** 10.3390/curroncol29030123

**Published:** 2022-02-27

**Authors:** Mary Jane Esplen, Jiahui Wong, Mary L. S. Vachon, Yvonne Leung

**Affiliations:** 1 Department of Psychiatry, Faculty of Medicine, University of Toronto, Toronto, ON M5T 1R8, Canada; jiahui.wong@desouzainstitute.com (J.W.); maryvachon@sympatico.ca (M.L.S.V.); yvonne.leung@desouzainstitute.com (Y.L.); 2 de Souza Institute, University Health Network, Toronto, ON M5T 1R8, Canada; 3 Dalla Lana School of Public Health, University of Toronto, Toronto, ON M5T 1R8, Canada; 4 College of Professional Studies, Northeastern University, Boston, MA 02115, USA

**Keywords:** grief and loss, compassion fatigue, resilience, burnout, educational program, health professionals

## Abstract

Health professionals working in oncology face the challenge of a stressful work environment along with impacts of providing care to those suffering from a life-threatening illness and encountering high levels of patient loss. Longitudinal exposure to loss and suffering can lead to grief, which over time can lead to the development of compassion fatigue (CF). Prevalence rates of CF are significant, yet health professionals have little knowledge on the topic. A six-week continuing education program aimed to provide information on CF and support in managing grief and loss and consisted of virtual sessions, case-based learning, and an online community of practice. Content included personal, health system, and team-related risk factors; protective variables associated with CF; grief models; and strategies to help manage grief and loss and to mitigate against CF. Participants also developed personal plans. Pre- and post-course evaluations assessed confidence, knowledge, and overall satisfaction. A total of 189 health professionals completed the program (90% nurses). Reported patient loss was high (58.8% > 10 deaths annually; 12.2% > 50). Improvements in confidence and knowledge across several domains (*p* < 0.05) related to managing grief and loss were observed, including use of grief assessment tools, risk factors for CF, and strategies to mitigate against CF. Satisfaction level post-program was high. An educational program aiming to improve knowledge of CF and management of grief and loss demonstrated benefit.

## 1. Introduction

Caring for patients with cancer is highly rewarding, attracting health professionals to the field who enjoy the challenge of managing a complex and life-threatening illness. Cancer care providers often form close bonds with their patients while providing long-term support to help them confront ongoing disease or treatment impacts [1,2]. Providers of cancer care must also deal with high mortality rates [1]. Thus, exposure to suffering, dying, and death is common [2]. Longitudinal exposure to suffering and loss may contribute to an overwhelming sense of grief [3]. Medland et al. [4] noted that healthcare providers may ignore feelings of grief and if unrecognized or unaddressed, such feelings can become chronic and cumulative. Experiences with cumulative grief and loss may contribute to the development of compassion fatigue (CF), a term used to describe the physical and emotional reactions as a result of caring for patients and bearing witness to pain and suffering [2,5]. Recently, CF has received increased attention within the current context of the COVID-19 pandemic [6]. CF and burnout have been linked to reduced work–life satisfaction, as well as high attrition and increased absenteeism [7,8]. Quality of care can also be impacted [7]. Zhang et al. [9], in a meta-analysis of 21 studies, found a prevalence rate of CF at 52.55% among nurses. A study of medical oncologists showed that 51.2% suffered from emotional exhaustion, 31.8% from depersonalization, and 6.8% from a lack of personal accomplishment [10]. Thus, supporting healthcare providers to manage their grief and loss experiences is an important factor to consider in maintaining the healthcare workforce.

### 1.1. Background

The significant impacts from caregiving in healthcare settings involving multiple patient loss and exposure to suffering have been well documented in the literature [5,11]. The term “wounded healer” has been used to refer to the suffering carried by the healer as they care for their patients [12,13,14]. Houck [15] emphasized the importance of recognizing grief and loss in practice as nurses in their “caring” roles grieve when patients die. When grief is acknowledged and supported, nurses are more able to cope through the finding of meaning in the loss [15]. Others have suggested that the recognition of grief experiences facilitates the delivery of compassionate patient care and healthy relationships [16]. In contrast, if experiences with loss and grief are left unrecognized (disenfranchised grief), difficulties with coping can occur [2,15]. Symptoms of cumulative grief may include physical illness, substance abuse, suicidal thoughts, apathy, poor self-esteem, depression, and anxiety [15]. Related reactions can involve a pattern of emotional detachment from patients or the overinvestment in patient’s lives, which can lead to the development of CF [17,18]. 

Compassion fatigue (CF) is defined as a specific tension related to caring, which occurs through the re-experiencing of traumatic events and persistent arousal associated with the patient’s suffering and distress [18,19]. Common symptoms include chronic exhaustion, reduced feelings of empathy, dreading working for or taking care of another, and feeling guilty thereafter [18,19]. CF has previously been thought of in association with an empathic process, known as *disruption in empathy* [20]. Wilson and Lindy [21] described CF as an “intrusive” empathic strain between the clinician and client that can result in over-identification. For example, empathic strain is characterized by being distant and avoiding contact with the patient. These two states are not empathic in the therapeutic relationship; rather, they can be viewed as dysfunctional processes [22,23]. 

Many factors contribute to the development of CF, including lack of knowledge about CF, lack of support, and being unable to ease suffering [24]. Perez et al. [25], in a qualitative study of palliative care practitioners, identified three main areas of stressors for CF: (1) systematic challenges related to managing large, emotionally demanding caseloads within time constraints; (2) patient factors (e.g., meeting patient/family demands); and (3) personal challenges of maintaining emotional and professional boundaries. These stressors challenge a professional’s effort to provide the desired level of care and may contribute to feelings of guilt and a sense of diminished competence [25]. 

Protective factors have been identified and include peer support, sense of competence in work-related tasks, work-life balance, connection and compassion towards self and others, specific knowledge about CF and risk factors, maturity/experience, and educational level [9,20,24]. Knowledge and skill in self-care have been identified as important competencies in practice [26]. However, health professionals often have little knowledge on the topic of CF, or access to interventions to support resilience and the management of impacts related to exposure to suffering and losses in their practice [24,27]. Further, personal barriers such as shame, stoicism, and feelings of personal failure can impede the reaching out for assistance [28,29].

A variety of intervention programs to prevent and manage CF and the impact of patient loss have been described [30]. Strategies include self-care wellness and education interventions [30]. Opportunities for group de-briefing sessions that incorporate rituals can assist health professionals to come together as a team and to share in the expression around a loss, contributing to a sense of meaning in the work [31]. Other programs have utilized resources such as mental health specialists to provide support [17], the teaching of mindfulness stress reduction [32], and strategies to enhance skill in self and other compassion [23,27,29,33]. 

In relation to educational interventions, Meadors and colleagues [34] developed an educational module to support self-care. Topics included CF definitions, symptoms, and strategies for reducing clinical stress. The program was evaluated, demonstrating benefit among healthcare providers working in pediatric intensive care units [34]. Other educational programs, such as the Compassion Fatigue Resiliency program is based in part on the Accelerated Recovery Program (ARP). It includes a 4-hour training program providing information for recognizing CF symptoms and triggers, the identification of resources for countering CF, and skill-building to address the physical, behavioral, and psychological demands while working in healthcare settings [35]. 

### 1.2. Objective

The objective of the project was to evaluate the impact of a continuing education program (“Managing Grief and Loss Amongst Healthcare Professionals”) on knowledge and confidence among oncology staff who have repeated exposure to suffering and loss, CF, and burnout. Kirkpatrick’s training evaluation model was used for the evaluation [36]. We also assessed the course impact on strategies that support the management of grief and loss following the education program. 

## 2. Methods

A continuing educational program on CF and managing grief and loss was developed and delivered by de the Souza Institute via a virtual classroom. The de Souza Institute is a national educational institution in Canada that provides online continuing education courses to healthcare providers, covering core competencies in oncology care. Curriculum topics range across the cancer care trajectory to support prevention, acute, psychosocial, and palliative care. The management of grief and loss offering has the objectives of providing education and support towards self-care of the healthcare provider. All de Souza courses integrate best practices and clinical guidelines to support quality improvement (QI) initiatives, and therefore, research ethics approval was not required for the evaluation. The program was offered as a part of a menu of offerings (requiring registration) with educational credits and was listed on the de Souza website and in its course calendar. It was open to all participants and not per se a required course specific to a particular work setting. This report includes evaluation of participants who received the program from 2011 to 2019.

### 2.1. The Educational Intervention

The primary aim of the program was to increase knowledge and confidence related to the impacts of exposure to suffering and loss, including understanding CF and burnout and to support the recognition of symptoms and types of grief experiences. 

### 2.2. Format

Participants attended weekly 1.5 h videoconference-based group sessions over six weeks. Each seminar included readings from the literature and was led by a PhD-level practitioner/educator with experience in grief/loss and psychotherapy. Weekly discussions were supported with an online community of practice (CoP). The CoP was included as an added source of resilience as it provided a reflective space with group members (who were enrolled in the same offering of the course) for recognizing and sharing experiences of grief and loss, their difficult cases, and resources. The shared knowledge and group support can be a source of strength and benefit to increase confidence and resilience of participants [37].

### 2.3. Content

The content of the course was organized in relation to the following: literature and a summary of personal, healthcare system/organization, and team-related (interpersonal) risk and protective factors associated with CF and burnout. Content on specific and evidence-based strategies to support coping and resilience and the management of grief and loss was also provided and explored. Strategies reviewed ranged from individual-oriented wellness strategies (e.g., psychotherapy, counselling, health-related behaviors), to team-level strategies (e.g., rituals that a clinical unit could employ to support meaning around loss and coping, group retreats or support programs; team grief debriefs; team-based compassion strategies) and organizational-level (e.g., review of patient volume/workload; managers checking in with staff and including review of well-being during annual performance reviews; management interventions supporting staff who recently endured traumatic loss; communication strategies; ensuring staff attend/identify wellness programs) (see Table 1).

The first three sessions focused on a review of grief models and reactions, and definitions of burnout and CF. Personal risk factors associated with CF were reviewed, as well as potential contributing risk or protective factors related to work settings or the team. Participants were encouraged to have an understanding of a theoretical model of grief to help guide them in their clinical practice as they encountered patients/families experiencing grief symptoms. Grief models were considered in relation to assessment of symptoms, levels and severity of grief reactions, phases/or stages of grief experiences, and how to support grief management. While the application of theory discussions focused on providing care to families experiencing anticipation grief or experiences of loss following a death, the discussions also included how participants might consider their own emotional experiences or physical symptoms as they encountered and experienced loss. As an example, readings were provided on the Dual Process Model of grief [38]. The topic of Medical Assistance in Dying (MAID) was also included.

The final three sessions focused on strategies and resources known to facilitate coping and that mitigate against CF and burnout. Participants used knowledge gained in the course to develop personal plans to take forward post-course, which incorporated strategies that “they could do” to support their own well-being, the work environment, or their team. Participants were encouraged to identify and consider how to address any relevant personal factors that emerged in their learning. In the final class, participants shared their plans and brainstormed around potential barriers associated with their implementation and strategies to address potential issues. 

The Revised Grief Experience Inventory (RGEI) [39] was given at baseline as a self-assessment tool and also incorporated as a teaching aid. For example, the program leader reviewed the tool’s items to illuminate specific domain areas for the assessment of grief reactions and for guidance in its application for assessing patients or caregivers. This exercise provided a type of mirroring experience, as participants reflected on personal experiences related to loss, while considering application of the tool in their clinical work. 

In addition, participants were asked to post the description of a “difficult case” that felt unresolved to them. Case narratives were utilized in class discussions to illustrate variables (e.g., patient variables, health professional variables, work-setting factors) that may have played a role in the sense of a lack of resolution. Participants were invited to reflect upon their cases throughout the course, applying gained understanding of relevant factors that may have played a role, and to consider what “they might do differently, if a similar situation presents once again”.

## 3. Evaluation Components

### 3.1. Demographics

A demographic questionnaire collected information on age, sex, profession, clinical setting, percent of cancer patients case load, number of patient deaths over the past year, and number of significant losses in the participant’s personal life over the past five years.

### 3.2. Grief

To assess psychosocial functioning in relation to grief symptoms, the Revised Grief Experience Inventory (RGEI) was given prior to the first session. The 22-item inventory has high internal consistency reliability (coefficient alpha 0.93) and consists of four domains: depression, existential concerns, guilt, and physical distress. The scoring is based on a self-report Likert-scale style with five possible responses ranging from strongly disagree (1), to strongly agree (5). The RGEI subscales have reliability alphas of 0.87, 0.80, 0.72, and 0.83, respectively, and an overall internal consistency alpha coefficient of 0.93. It does not have a cut-off point or threshold for severe grief response. The instrument has been utilized in assessment of family caregivers [39] and health professionals [40].

### 3.3. Kirkpatrick Model for Evaluation of Training Programs 

Using the Kirkpatrick training evaluation model [28], we evaluated the reaction/satisfaction and learning/knowledge of an educational program. A pre- and post-course survey (within 1 week of the course ending) was completed by participants.

*Level 1 Reaction—Satisfaction with the program:* The satisfaction was assessed by a 5-point Likert scale after each session, with 5 being “Strongly agree”, on quality of course content, presentation, discussion, and whether the session met learning needs. Two open-ended questions in the post-course survey were used to gather qualitative feedback, including asking participants to identify two important learnings that they took away from the course and to provide feedback on which components they particularly valued.

*Level 2 Learning: Knowledge and Confidence in Managing Grief and Loss:* A self-report questionnaire was developed by the evaluation team and lead course educators with content expertise and included items linked to learning objectives of the program. The survey was given pre- and post-course and consisted of 13 items with a 4-point Likert-type scale from “Not knowledgeable at all” (1), “Not very knowledgeable (2), “Somewhat knowledgeable (3) to “Very knowledgeable” (4). Item content included five domains: (1) Recognizing the Signs of burnout and CF and its Impacts; (2) Factors Contributing to the Ability to Manage Loss; (3) Reflections on Experiences of Loss and Grief; (4) Strategies that Healthcare Professionals can Integrate into their Practice to Support Management of Grief and Loss; and (5) Team and Organizational-based Strategies. A single item 10 cm visual analogue scale (VAS) was used to assess participants’ confidence in the “ability to recognize and manage your own grief and loss”, anchored with “Not confident at all” to “Very confident” and was given pre- and post-program.

*Level 3 Behaviors: Personal Plans:* Participants posted and discussed personal plans in the final sessions of the course. They were also asked to rate their intention to carry out specific behavioral changes, including (i) apply self-assessment tools to identify loss and grief; (ii) facilitate discussions with colleagues on issues of grief management and form a peer group to obtain support for each other; (iii) communicate to supervisor about personal experiences with grief and loss; (iv) apply various stress management techniques to reduce distress and enhance coping and wellness; and (v) seek support from a mental health professional when having difficulties in managing grief. 

## 4. Evaluation Analysis 

A descriptive analysis was conducted. A stepwise regression analysis examined the association between demographic variables (age, sex, health profession, amount of loss in work setting and in personal life) and the baseline RGEI to understand current functional level in relation to grief symptoms. A paired t-test was conducted (pre-post survey) to assess the impact of the program on confidence and knowledge measures. When handling missing items within a measure, prorated scores were used if respondents had <20% of the items missing. A content analysis was conducted by the evaluation team using NVivo to explore content of open-ended questions and participant personal plans. 

## 5. Results

### 5.1. Participants

A total of 189 healthcare professional learners enrolled into the program between 2011 and 2019. There were no dropouts from the course. The majority of participants were nurses and female (92.6%) (see Table 2). Approximately 16% of participants were younger than 29 years, 49% were between 30–49 years of age, and 32% were 50 or older. Participants reported a mean of 17.7 years of clinical practice, of which 7.3 years were solely in oncology settings. Approximately 38% worked in cancer centers, 23% in palliative care, 21% in community care, and 18% in a general hospital unit. Most participants were direct care clinicians with 10% working as educators or managers.

The distribution reflects the well-documented cancer journey involving community care, highly specialized cancer care, and/or palliation. There were only four participants who had no cancer patients in their practice. They include a manager in occupational health supporting providers in cancer care. The sample also includes Educators/Managers who may not have direct care role but are supervising and supporting their staff involved in cancer care. 

In relation to loss, 59% of participants experienced 10 or more patient deaths in a typical year and 12.2% were exposed to more than 50 patient losses per year (See Table 2).

### 5.2. Healthcare Provider’s Grief Experience 

The original RGEI does not have a cut-off score for grief experience. Using one standard deviation above the mean in our program to identify individuals who demonstrated significant grief symptoms, 18% of participants met the criteria (see Table 3). The total grief symptom score was not associated with demographic variables, the percent of cancer patients in the caseload, or the number of deaths in the previous 12 months of participants’ work experience. 

In considering the RGEI total score and subscales, comparing it with the literature, the mean for participants in the program was significantly lower than that reported for primary family caregivers of a dying patient [39] in both total score and subscales. However, they were significantly higher than that reported among a sample of pediatric nurses [40] (Table 3).

### 5.3. Kirkpatrick Model for Evaluation of Training Programs 

*Level 1 Reaction:* Satisfaction with the program was high (85% scored >=4 on average; range 1–5) in all four areas of quality of course content, presentation, discussion, and whether the session met learning needs. Several participants (89%) suggested that this type of program should be provided earlier in their careers or during formal education (e.g., *“This program should be encouraged during our education”; “I wish I had known all this earlier in my career”*). Other participants expressed surprise at their gained insight concerning the role of personal factors (e.g., *“I had no idea that my own personal factors could be playing so much of a role”; “I didn’t realize how little time I was taking to take better care of me; “It’s normal to not have* *everything work out well each time”*). The group format was also reported as an ideal set-up (94% of participants).

**Figure 1:**
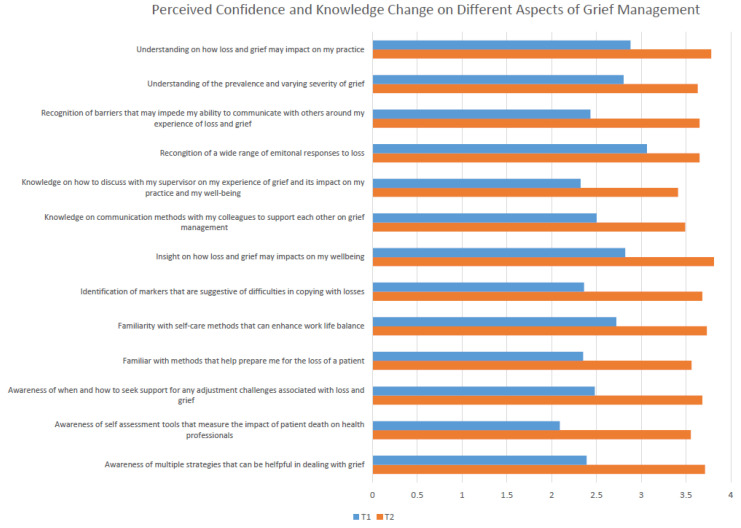
Perceived knowledge change. (1 = Not knowledgeable at all to 4 = Very knowledgeable).

*Level 2 Learning:* At baseline, the total score on confidence and knowledge as measured by the 13 statements was 35.30 (±12.01) on average, ranging from 13 to 52. Post-course, the total mean score increased to 41.06 (±16.60) and the increase was statistically significant (t(188) = 4.02, *p* < 0.05). The average score for individual statement was 2.71 (±1.08). This average score represents a knowledge level just above “Not very knowledgeable (2)” but below “Somewhat knowledgeable (3)”. The average score for individual statement increased to 3.64 (±0.50) post-course, representing a knowledge level approaching “very knowledgeable (4)”. Paired t-test analysis indicated statistically significant improvement in all 13 items (See Figure 1). A change from pre- to post-intervention on the single item (0 to 10) (VAS) assessing participant confidence in being able to recognize and manage grief and loss was also significant. Participants reported an average score of 6.9 (±1.6) at baseline, which increased to 9.2 (±1.0) post-program (t = 16.21, *p* < 0.01).

**Figure 2:**
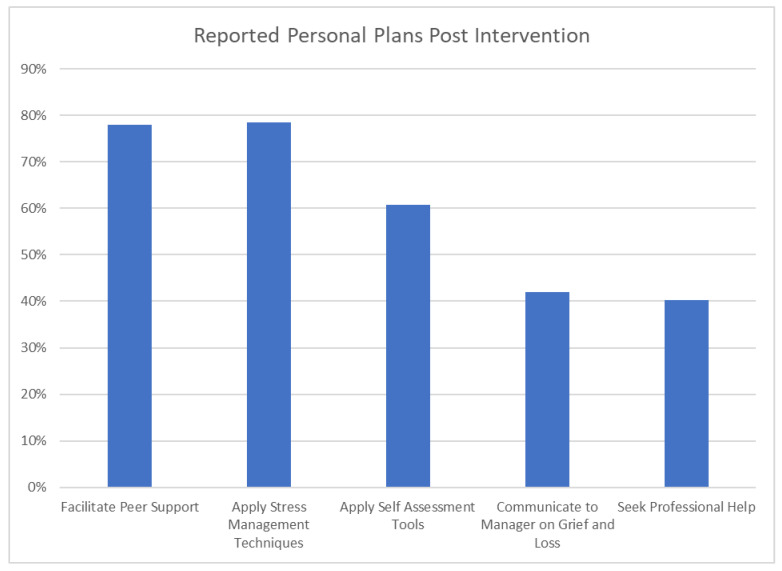
Strategies and personal plans to manage CF post intervention.

*Levels 3 Behaviors:* Participants described specific strategies in their personal plans during the last sessions of the course and on post-course feedback. The most common strategy reported comprised of plans to facilitate peer support through increased discussions on CF with colleagues (78%) and the organization of a peer support group (78%). Plans to use a self-assessment tool to assess grief or symptoms associated with CF (61%) and to use strategies to support personal coping and stress management (78%) were also common. Finally, 40% of participants indicated that they plan to seek professional help when necessary (See Figure 2).

## 6. Discussion 

Prior to entering the course, participants demonstrated a low level of knowledge and confidence concerning the topic of grief and loss and CF, as well as in being able to identify strategies to support coping with exposure to suffering and loss in clinical practice. The program outcomes demonstrated what we consider to be significant benefit. Improvements in knowledge and confidence related to a number of domain areas were observed, including in the identification of risk factors or indicators for CF and burnout, and knowledge of grief reactions and patterns, as well as personal self-care or team-based strategies that can be helpful to support resilience and assist in the management of grief and loss. 

Interestingly, most participants reported that the course was the first opportunity to learn about their own personal risk factors that can play a role in managing loss and grief or that contribute to CF. The majority of participants (90%), upon entering the program, believed that contributing factors to difficulty in coping with loss or CF were primarily related to work setting (e.g., patient volume, time constraints, or workload). Participants over time gained a comprehensive and broad view of CF, burnout, and contributing factors. While perhaps less known, a health professional’s personal factors are also relevant, particularly given the role of the “self” involved in the healthcare provider/patient relationship that forms while providing supportive and psychosocial care [41] with oncology populations and in palliative care [42]. 

Participants reported experiencing considerable loss in their practice settings. Fifty-nine percent of participants experienced 10 or more losses in a typical year and 12.2% experienced more than 50 annually. Despite having practices that frequently involved loss of patients, it was surprising to find that more than 80% of the mainly nurse participants were unfamiliar with a model of grief. While it is likely that participants had received education on theories of grief during their formal educational preparation, this finding suggests a need for continuing education to keep pace with the empirical and theoretical literature on grief and loss.

In considering the RGEI score, the overall mean for participants in the program was lower than that reported for primary caregivers (family members) of a dying patient. The observation is consistent with the literature [43], suggesting that nurses do experience grief over the death of their patients in ways similar to the patient’s family members, yet to lesser extent.

In relation to the Adwan et al., 2014 study of pediatric nurses, although pediatric death is a highly stressful experience to clinicians [43], the volume of deaths/year was lower in the pediatric study (39% reported 1 or more patient deaths) than many participants experienced in our program (59% reported 10 or more patient deaths). This factor may have played a role in the observed differences between our study and that of the pediatric nurses (Adwan et al., 2014). 

Participants in the program demonstrated a range of grief scores, indicating variation in emotional and physical reactions. However, a sub-group of participants (18%) demonstrated a relatively high level of grief symptoms, supporting the notion of the “wounded healer” [23] described in the literature. Several participants working in inpatient settings expressed strong emotions around cases that were traumatic in nature, especially around unexpected or untimely deaths (e.g., young patients). Fillion et al. [44] studied nurses working in end-of-life care and other settings in Quebec. Nurses working in critical care and oncology units reported high stress compared with specialized PC units [44]. 

Unfortunately, we did not include a standardized measure of CF or burnout, and therefore, we cannot observe if there are associations between grief scores and symptoms of burnout or CF. The literature suggests that health-professional grief, especially if cumulative over time, can lead to CF or burnout if not addressed [8,18]. Similar to findings in the literature, participants in this program (50%) reported a coping response of maintaining a need to ”carry on” [24,28] and a stance of not expressing emotion. Some participants described the challenge of having to prepare a room for the next admission immediately following a death of a patient. 

Only one-quarter of participants (24%) reported having either a formal or informal opportunity to address their experiences around loss in their settings. Similar to the literature, opportunities for debriefs or rituals around patient deaths were reported more frequently among participants working in palliative care settings [25]; however, participants providing palliative care also reported lack of sufficient support. A significant number of participants reported time pressures (75%) and a lack of resources in bringing the team together to explore issues in their work (50%) as contributing to work strain. 

More than 88% of participants reported that work-related well-being is not raised during their annual reviews. These findings are consistent with the literature and underline a work culture focused on patient and system outcomes [28]. Findings may also reflect personal and/or system barriers (e.g., healthcare training socialization and healthcare culture) that may make it difficult for healthcare providers to express coping challenges among peers or to supervisors [24,28]. In a qualitative study of oncologists, participants noted that the interviews were the first time they were asked about their coping in relation to their work [28].

The COVID-19 pandemic has highlighted the need for the provision of wellness programs for staff and for careful consideration of healthcare system and team issues that can contribute to burnout and CF [6]. Health professionals in this program welcomed the opportunity to reflect upon and share with their peers on cases and specific factors that may have contributed to their difficulty in coping or resolving issues associated with a case. 

The group classroom discussions and community of practice were well received. Comments such as, *“Seeing others having similar issues was surprising”;” I learned so much from them”* support a group-oriented approach to facilitate normalization, open expression, and vicarious learning that may reduce stigma in expression of personal experiences. Several participants also expressed valuing course instructors sharing experiences, *“I appreciate the leader disclosing, sharing and the openness of the forum”*.

## 7. Limitations

The majority of participants were nurses and female, and therefore, program outcomes may not be generalizable to other health providers or genders. We also did not include measurement of CF in the evaluation, so we were unable to assess the program’s impact on specific symptoms or levels of CF. The program evaluation focused on short-term outcomes, and therefore, we are unable to assess long-term benefits by conducting follow-up assessment on the actual implementation of participants’ proposed plans. 

## 8. Conclusions

In summary, the program evaluation indicates that a fairly brief continuing education program, focusing on managing grief and loss, is beneficial in improving knowledge and confidence in relation to identifying symptoms of CF and burnout, risk factors for CF, and strategies that can help support resilience and manage exposure to loss and suffering in clinical practice. The program also helped guide individuals to develop a personal plan to address specific difficulties or issues that they identified through the course content relevant to their personal background/coping or to work environment. The group format and the topics related to personal, team-related, and organizational contributing factors to CF were well received. Participants left the program with plans to support their work settings and self-care. Further work is needed to assess longer-term benefits of the program. 

## Figures and Tables

**Table 1:** Description of the educational program.

Session	Topics	Course Strategies
Session I	Burnout and compassion fatigue Definitions Prevalence Symptoms	Posting of “difficult” case that felt unresolved; Sharing/discussion Assigned readings Sharing in community of practice
Session II	Risk and contributing factors Health professional personal variables Patient variables Team and work environment factors	Lecture Assigned readings Completion of RGEI (Revised Grief Experience Inventory) Discussion Sharing in community of practice
Session III	Review of grief models Theory Grief symptoms Assessment Importance of having an “understanding” of a theoretical model of grief and loss	Lecture Assigned readings Discussion Sharing in community of practice
Session IV	Strategies to support resilience/address compassion fatigue; prevention	Lecture Assigned readings Discussion Sharing in community of practice
Session V	Strategies to support resilience/address compassion fatigue; prevention	Lecture Assigned readings Discussion Sharing in community of practice
Session VI	Reconsideration of posted cases from week 1; Personal plans going forward (e.g., personal well-being, team-oriented plan; organization-oriented plan)	Review of personal plans; Discussion and identification of barriers/enablers and strategies to address them Discussion re posted case in week 1

**Table 2:** Participant characteristics (*n* = 189).

Variables	N	%
**Age**		
<29	30	15.93
30–49	92	48.7
50+	61	32.3
No answer	6	3.2
**Sex**		
Female	175	92.6
Male	7	3.7
No answer	7	3.7
**Profession**		
RN	173	91.5
Other (i.e., social workers, radiation therapists, occupational health)	16	8.5
**Clinical Setting**		
Cancer Centre, Cancer Clinic	72	38.1
Hospice Palliative Care	43	22.8
Home Care, Community Care	40	21.1
General Hospital	34	18.0
**Percent of Cancer Patients**		
All are cancer patients	64	33.9
More than half	55	29.1
A third or less	54	28.5
None	4	2.1
No answer	12	6.4
**Seeing a Grief Therapist**		
Y	46	24.3
**Number of Patient Deaths in previous year**		
<10	66	34.9
10–29	69	36.5
30–49	19	10.1
50+	23	12.2
No Answer	12	6.4
**Personal Loss (5 years) (answers not mutually exclusive)**		
Loss of a close family member	57	30.1
Loss of a friend	62	32.8
Loss of a relative/co-worker	51	27.0
Loss of a pet	35	18.5
No loss	30	15.9

**Table 3:** Revised Grief Experience Inventory (RGEI) score.

	Lev’s Study with Primary Care Givers [39] (N = 418)	Adwan’s Study with Pediatric Nurses [40] (N = 120)	Current Educational Intervention Participants (N = 189)
Total score mean (SD)	75.5 (25.7)	48.3 (17.8)	60.52 (25.1)
Range	22–132	22–94	22–126
Subscales			
Depression	23.0 (7.0)	14.9 (7.6)	19.13 (7.7)
Physical Distress	22.5 (9.3)	15.1 (6.3)	18.34 (8.2)
Existential Concerns	20.1 (8.5)	11.1 (4.8)	14.5 (7.8)
Tension and Guilt	10.0 (4.6)	7.3 (2.8)	8.6 (3.9)

Note: Although not as high as primary family care givers in palliative care, participants in the de Souza course scored higher than pediatric nurses in Adwan’s study; 18% of participants had total scores >85.5 (de Souza course mean score +1SD); 26% had scores >75.5 (average total score Lev’ primary care giver study [39]); 26% had seen a grief counselor prior to the course, which could be for any loss and which may or may not be related to work.

## Data Availability

Data that support the findings of this study are available on request from the corresponding author.
